# Mechanisms of tilt‐induced vasovagal syncope in healthy volunteers and postural tachycardia syndrome patients without past history of syncope

**DOI:** 10.14814/phy2.14148

**Published:** 2019-06-27

**Authors:** Julian M. Stewart, Mohamed A. Shaban, Tyler Fialkoff, Brianna Tuma‐Marcella, Paul Visintainer, Courtney Terilli, Marvin S. Medow

**Affiliations:** ^1^ Departments of Pediatrics and Physiology New York Medical College Valhalla New York; ^2^ Baystate Medical Center University of Massachusetts School of Medicine Springfield 01199 Massachusetts

**Keywords:** Cardiac output, false positive Tilt, POTS, vasoconstriction, vasovagal syncope, young

## Abstract

Upright tilt table testing has been used to test for vasovagal syncope (VVS) but can result in “false positives” in which tilt‐induced fainting (tilt+) occurs in the absence of real‐world fainting. Tilt+ occurs in healthy volunteers and in patients with postural tachycardia syndrome (POTS) and show enhanced susceptibility to orthostatic hypotension. We hypothesized that the mechanisms for hypotensive susceptibility differs between tilt+ healthy volunteers (Control‐Faint (*N* = 12)), tilt+ POTS patients (POTS‐Faint (*N* = 12)) and a non‐fainter control group of (Control‐noFaint) (*N* = 10). Subjects were studied supine and during 70° upright tilt while blood pressure (BP), cardiac output (CO), and systemic vascular resistance (SVR), were measured continuously. Impedance plethysmography estimated regional blood volumes, flows, and vascular resistance. Heart rate was increased while central blood volume was decreased in both Faint groups. CO increased in Control‐Faint because of reduced splanchnic vascular resistance; splanchnic pooling was similar to Control‐noFaint. Splanchnic blood flow in POTS‐Faint decreased and resistance increased similar to Control‐noFaint but splanchnic blood volume was markedly increased. Decreased SVR and splanchnic arterial vasoconstriction is the mechanism for faint in Control‐Faint. Decreased CO caused by enhanced splanchnic pooling is the mechanism for faint in POTS‐Faint. We propose that intrahepatic resistance is increased in POTS‐Faint resulting in pooling and that both intrahepatic resistance and splanchnic arterial vasoconstriction are reduced in Control‐Faint resulting in increased splanchnic blood flow and reduced splanchnic resistance.

## Introduction

Syncope is defined by rapid onset transient loss of consciousness and postural tone due to cerebral hypoperfusion with spontaneous recovery (Moya et al. 2009). Most of syncope in young patients is due to simple faint, denoted “vasovagal syncope” (VVS) (Sheldon et al. 2015), often triggered by upright posture. VVS is quite common in young people with an incidence of one or more events occurring in approximately 20–40% of the general population (van Steenwijk et al. 1995; Ganzeboom et al. 2003). The diagnosis of VVS can often be made by taking a detailed history, obtaining an electrocardiogram and performing a detailed physical exam to rule out potential contributory cardiac disease and orthostatic hypotension (Freeman et al. 2011; Wieling et al. 2015). VVS can occur in patients diagnosed with the postural tachycardia syndrome (POTS). The incidence of VVS in POTS patients is believed to be about 30%, similar to its incidence in the general population (Raj 2006; Kimpinski et al. 2012), which is consistent with our experience.

Upright tilt table testing has been used in the diagnosis of VVS. Caution is advised regarding interpretation because tilt testing can fail to provoke syncope in some young patients with clinically confirmed real‐world VVS, but can also result in VVS during tilt in many healthy young volunteers with no history of fainting in the real world (Fouad et al. 1993; van Steenwijk et al. 1995; Lewis et al. 1997). This latter group has been often denoted “false positive fainting” (Leonelli et al. 2000). We have observed similar percentages of “false positive” faints in POTS patients during 10‐min unmedicated upright tilt testing at 70°.

“False positive” tilt tests have been reevaluated as demonstrating “hypotensive susceptibility” to orthostatic stress which potentiated syncope of whatever origin (Sutton and Brignole 2014) via reduced cardiac venous return (Verheyden et al. 2008; Jardine et al. 2018) or reduced systemic vascular resistance in younger patients with VVS (van Steenwijk et al. 1995; Stewart et al. 2017a; Jardine et al. 2018).

POTS is related to absolute or redistributive central hypovolemia with reduced venous return (Stewart and Montgomery 2004). We recently showed that hypotensive susceptibility in young healthy volunteers is driven by reduced SVR, the predominant mechanism of VVS in the young (Stewart et al. 2017a). In the present study, we therefore hypothesize that mechanisms for hypotensive susceptibility in POTS is likely related to decreased venous return and cardiac output.

To test this hypothesis, we examined hemodynamics in POTS and healthy volunteer controls. Neither POTS patients nor controls had prior history of VVS and both groups fainted during upright tilt. We also compared these data to healthy volunteers who did not faint during tilt testing.

## Methods

### Subjects

“False positive vasovagal syncope” enrollees comprised either POTS patients (designated POTS‐Faint) or healthy volunteer control subjects (designated Control‐Faint) free of past history of VVS but who developed vasovagal syncope during a 10‐min 70° upright tilt test. POTS patients had undergone an earlier upright 10‐min tilt test which confirmed the diagnosis of POTS including excessive tachycardia, symptoms of orthostatic intolerance (OI), and absence of hypotension.

VVS was identified by orthostatic prodromal features included pallor, lightheadedness, nausea with abdominal discomfort, diaphoresis, a feeling of warmth, visual scotomata or frank loss of vision, and loss of consciousness or impending loss of consciousness with hypotension and a relative bradycardia as defined below. Unconsciousness or impending loss of consciousness resolved in all participants within 30 sec when placed supine. All patients had normal resting electrocardiograms and resting physical examinations.

We prospectively enrolled 12 POTS‐Faint subjects aged 15–25 years old (mean age 20 ± 2 years, 10 females, 2 males) with POTS defined by standard criteria (Schondorf and Low 1993; Singer et al. 2011). Once POTS had been diagnosed previously by signs and symptoms of OI (Stewart et al. 2018) including an excessive increase in HR without hypotension within 10 min of a head‐up tilt during which they did not faint (Low et al. 1995; Raj 2006; Medow and Stewart 2007), patients were invited to enroll in this study. We only enrolled POTS patients categorized as “Normal Flow” to improve subject homogeneity, using our previously established techniques (Stewart et al. 2006). Symptoms of OI were present on a daily basis for >6 months and were relieved once supine. Excessive orthostatic tachycardia was defined in adolescents by an increase in heart rate (HR) by at least 40 bpm or to a HR > 130 bpm. Excessive orthostatic tachycardia was defined in patients over 19 years by an increase in HR by at least 30 bpm, or to a HR > 120 bpm, during a prior 10 min upright tilt table test (Freeman et al. 2011; Singer et al. 2011; Plash et al. 2013). Alternative medical or psychological problems that could explain these signs or symptoms had been ruled out. During participation in the present study, this subset of POTS subjects became syncopal during tilt table testing and is hence referred to as POTS‐Faint. We also prospectively enrolled 12 gender matched subjects aged 15–25 (mean age 21 ± 2 years, 10 females, 2 male) from a larger set of “false positive vasovagal syncope” healthy control subjects, designated Control‐Faint, from subjects who were evaluated for syncope.

We also prospectively recruited 10 healthy non‐fainting control subjects, with no past history of VVS or OI, aged 15 to 24 years (mean age 21 ± 1 years, 8 females, 2 males) for comparison with the two “faint” groups. These were designated Control‐noFaint. There were no differences in the ages, weight, and body mass index between groups. Since sex hormones influence cutaneous blood flow (Charkoudian and Johnson 1999), and systemic hemodynamics (Fu et al. 2010), all women in this study participated while in the early to midluteal phase, or high hormone phase of oral contraception, when autonomic tone is maximized (Minson et al. 2000).

Fainting patients did not meet the definition for orthostatic hypotension (Freeman et al. 2011) and were examined for cardiogenic causes of syncope; none were found. Control subjects reported no clinical illnesses, and had never previously fainted.

Exclusion criteria for participation in this study were any infectious or systemic disease (including other cardiovascular disease), recent long‐term bed rest, competitive athletic training, use of nicotine containing products or pregnancy within the last year. Medical therapy for POTS, if any, had been stopped for at least 2 weeks prior to participation in this study. Subjects refrained from caffeine for at least 72 h prior to testing. Subjects fasted for a minimum of 4 h prior to testing. This study was approved by the Institutional Review Board of New York Medical College. All subjects 18 or older signed an informed consent; those younger than 18 assented to participate and their parent or legal guardian signed an informed consent.

### Protocol

Subjects arrived at our center at 9:30 am. Tests and instrumentation were explained. Subjects were instrumented while supine. Beat‐to‐beat blood pressure was measured by Finometer finger photoplethysmograph (FMS, Amsterdam, The Netherlands) on the right forefinger or middle finger. The Finometer estimates beat‐to‐beat CO by pulse‐contour analysis using the Modelflow algorithm^®^ (Bogert and van Lieshout 2005). ModelFlow CO was calibrated against an Innocor inert gas rebreathing CO measurement (Innovision, Denmark) while supine before experiments began. We computed beat‐to‐beat systemic vascular resistance (SVR) by dividing the time average arterial pressure (mean arterial pressure, MAP) by the ModelFlow CO averaged over each cardiac cycle. We also computed the pulse pressure (PP) for each cardiac cycle by subtracting the diastolic from the systolic BP. Regional blood volumes, blood flows, and vascular resistance were measured by impedance plethysmography (Stewart et al. 2006). We placed paired electrodes using anatomic landmarks to estimate thoracic, splanchnic, pelvic, and calf segmental blood volumes. Respiratory plethysmography (Respitrace, NIMS Scientific, Miami Beach, FL) and capnography (Smith Medical PM, Waukesha, WI) measured changes in respiration and end tidal carbon dioxide (ETCO_2_). An electrocardiograph measured HR from the beat‐to‐beat cardiac electrical interval. Signals were acquired at 200 samples/s, multiplexed, and A/D converted using custom software.

Subjects remained awake and supine for 30 min to acclimate to instrumentation. Baseline data comprising averaged HR, BP, ETCO_2_, CO, SVR, and thoracic, splanchnic, pelvic, and calf segmental impedances and rate of change of impedances were collected. Baseline data over the 10 min immediately preceding tilt were used for comparison with tilted information.

Subjects were tilted upright to 70°. The duration of upright tilt was 10 min for Control‐noFaint. Fainting subjects remained upright until fainting was imminent which by design occurred within 10 min of tilt‐up. Tilt was performed without pharmacologic provocation. Continuous HR, BP, ETCO_2_, CO, SVR, and impedance data were recorded for off‐line analysis.

Fainting patients were tilted back to supine when syncope was imminent. Imminent vasovagal syncope was defined by a tilt‐induced decrease in mean arterial pressure (MAP) to <60 mmHg or a decrease in systolic BP (SBP) <70 mmHg associated with symptoms of impending loss of consciousness, severe lightheadedness, nausea, heat, or diaphoresis. Fainters developed classic vasovagal syncope with hypotension followed by bradycardia during tilt (Wieling et al. 2016; Stewart et al. 2017a; Jardine et al. 2018).

### Detailed methods

#### Fiducial event markers

**Figure 1:**
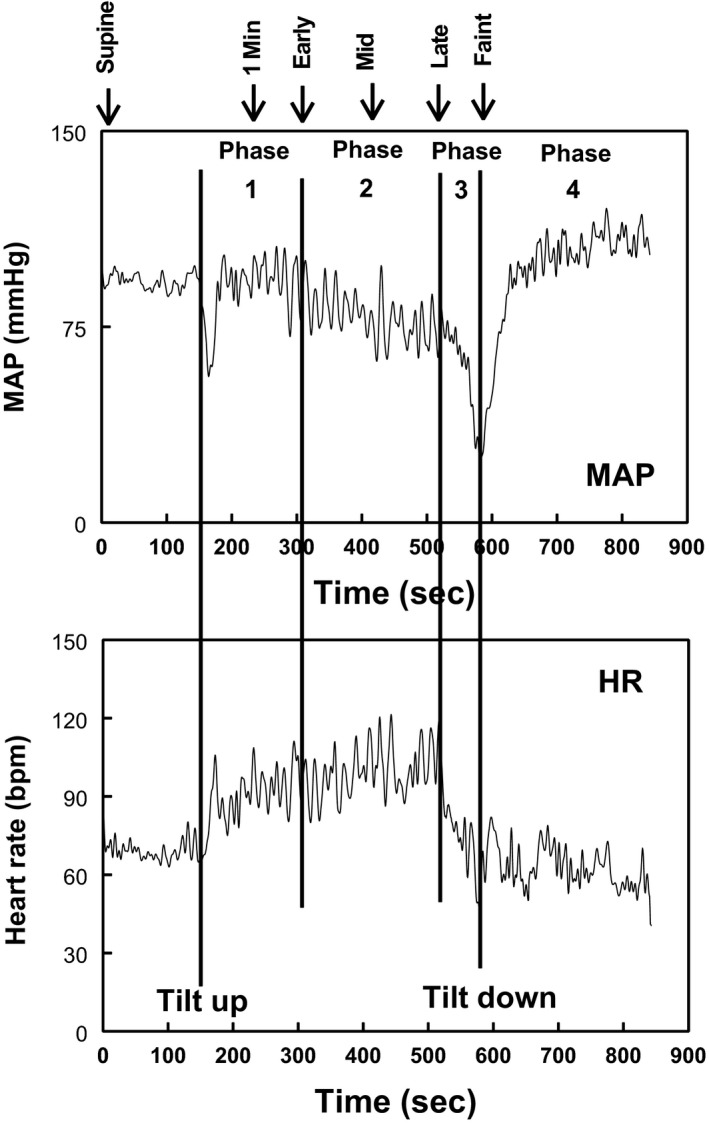
Head‐up tilt table test for a vasovagal syncope patient. Mean arterial pressure (MAP) is shown in the top panel and heart rate (HR) in the bottom panel. Fiducial points are marked at the top. MAP and HR follow a characteristic time course comprising four stages: Early blood pressure (BP) stabilization (Phase 1, fiducial point 2); “Progressive early hypotension” with BP slowly declining as HR increases (fiducial points 3–5); Syncope with hypotension and bradycardia (Phase 3, fiducial point 6). Phase 4 recovery of BP occurs within 30 sec once supine.

We analyzed results at fiducial time points, rather than at specific time points in accordance with previous work (Taneja et al. 2008; Stewart et al. 2017a). Fiducial markers identified comparable times at which physiological events occurred allowing us to study events which corresponded to similar occurrences in each fainter. Six defining events points were determined from the BP trace of each VVS subject; these events are represented for one subject in Figure 1, and correspond to the phases of vasovagal syncope described by Jardine et al. (2018). The first fiducial point, was baseline, denoted “supine” on the graph. Following initial orthostatic hypotension (Wieling et al. 2007), BP stabilizes (Phase 1); this is denoted “1 min” and was chosen to be mid Phase 1 approximately 1 min after tilt. Thereafter, a gradual progressive early hypotension associated with reflexively increased HR occurred and was identified with the onset of Phase 2 and is designated “early.” BP falls off rapidly and abruptly in Phase 3; Late phase 2 was identified at the transition between Phase 2 and Phase 3 and is designated “late.” We defined a “mid” point as midway between “early” and “late.” The last fiducial point, designated “faint,” occurred at the time of imminent syncope, or at 10 min for Control‐noFaint subjects. The non‐fainting control subjects did not have a significant fall in BP. We defined equivalent fiducial time points for Control‐noFaint by taking the averaged time of occurrence of each fiducial marker in fainting subjects divided by the time from onset of tilt (the fractional time to marker) and multiplied by 10. Control‐noFaint and fainting subjects were thus compared at equivalent times to obtain a uniform evaluation of hemodynamic quantities across groups.

#### Measurement of changes in segmental blood volumes

Changes in segmental or regional blood volumes and segmental blood flows employed impedance plethysmography (IPG) using a tetrapolar high‐resolution four‐channel digital impedance plethysmograph (UFI, Morro Bay, CA) to measure impedances and the rate of change of impedances as described previously (Montgomery 1993a,b). These quantities were obtained within four anatomic segments defined by electrode placement on anatomic landmarks that delimit respective regional circulations. These were designated the thoracic segment (supraclavicular area to xyphoid process), the splanchnic segment (xyphoid process to iliac crest), the pelvic segment including lower pelvis to the knee (iliac crest to knee), and the leg or calf segment (upper calf just below the knee to the ankle).

#### Measurement of changes in segmental blood flow

IPG was also used to estimate segmental or regional blood flows (Montgomery et al. 1993). These methods have been validated in our laboratory against the reference standard indocyanine green dye techniques and calf venous occlusion plethysmography and were used to measure leg, thoracic, and splanchnic blood flow while subjects were supine and during incremental tilt‐table testing, as described previously (Stewart et al. 2006, 2007).

### Data analysis and statistics

All data were digitized and stored and were analyzed off‐line with custom software. There were three subject groups for comparison: Control‐noFaint, Control‐Faint, and POTS‐Faint.

Baseline data for BP, HR, CI, SVR, and ETCO_2_ are shown in absolute units in Table 1. We also analyzed time to faint for both groups. These were compared among the groups by one‐way ANOVA. Significant interactions if any were further analyzed by converting the ratio of *F* values to a *t‐*distribution by use of a Scheffé test, and probabilities were determined thereafter. All tabular results are reported as mean ± SEM.

**Table 1:** Baseline hemodynamics

	Control – No Faint	Control – Faint	POTS – Faint
Systolic BP (mmHg)	116 ± 4	120 ± 3	121 ± 4
Diastolic BP (mmHg)	61 ± 3	62 ± 3	65 ± 2
MAP (mmHg)	79 ± 2	83 ± 3	83 ± 3
HR (bpm)	64 ± 3	66 ± 2	73 ± 3^a^
CI (L/min/m^2^)	4.8 ± 0.5	5.3 ± 0.3	5.5 ± 0.4
SVR (mmHg/L/min)	18 ± 2	15 ± 2	17 ± 1
ETCO_2_ (Torr)	42 ± 2	42 ± 1	44 ± 1

a *P* < 0.05 compared to Control‐noFaint.

Graphic data are shown as absolute units for CI and SVR and as percent of baseline for segmental blood flows and resistance expressed as mean ± SEM. Data were obtained from original time series averaged over 15s intervals centered at the fiducial markers. Repeated‐measures ANOVA were used to compare groups at times before Faint. We assigned greatest importance to “group × time effects” representing the interaction of subject group with time dependent changes during upright tilt. We assumed a covariance structure of compound symmetry. Reported *P*‐values reflect the interaction term using the Greenhouse‐Geisser correction. Statistical significance was set at *P* ≤ 0.05. Results were calculated by using GraphPad Prism version 8. Significance (*P*‐values) appears in the figures as well as text.

## Results

### Baseline supine data

Baseline data measured while subjects were supine are displayed in Table 1, which shows data for Control‐noFaint, Control‐Faint, and POTS‐Faint. There were no baseline differences in systolic, diastolic or mean BP, CI, SVR, or ETCO_2_ between Control‐noFaint and fainting groups. HR was significantly higher in POTS‐Faint compared with Control‐noFaint (*P* < 0.025).

### Upright tilt data

#### Time to faint

The time from tilt up to syncope for Control‐Faint averaged 375 ± 49s, and was not different from the time to syncope for POTS‐Faint which averaged 349 ± 58 sec. By definition, Control‐noFaint subjects did not experience symptoms of orthostatic intolerance nor did they faint.

#### Representative tracings during tilt, VVS groups

**Figure 2:**
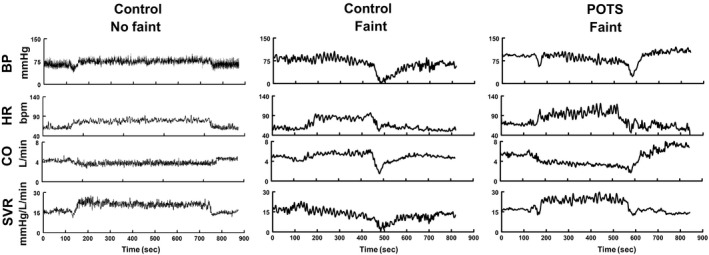
Blood pressure, heart rate, cardiac output, and systemic vascular resistance for representative subjects during head‐up tilt table testing. Figure 2 shows representative data from left to right for Control‐noFaint, Control‐Faint and POTS‐Faint subjects. Each data panel contains graphs of BP, HR, CO, and SVR in top to bottom order for representative subjects belonging to each group. HR increased in all groups during orthostatic stress. Following initial pressure stabilization there was a gradual fall off in BP in fainters representing Phase 2. During Phase 2, CO increased above baseline for Control‐Faint while SVR decreased and CO progressively decreased throughout tilt in POTS‐Faint while SVR increased. Phase 3 ensued with rapid hypotension followed by bradycardia associated with decreased SVR and CO in all fainters.

Figure 2 shows BP, HR, CO, and SVR for representative subjects belonging to each group. HR increased in all groups during orthostatic stress imposed by an upright tilt to 70°, shown by the arrows. Following initial BP stabilization there was a gradual fall off in BP in both Control‐Faint and POTS‐Faint representing Phase 2. During Phase 2 CO increased above baseline for Control‐Faint while SVR decreased. During Phase 2 CO progressively decreased throughout tilt in POTS‐Faint while SVR increased. Phase 3 ensued in both Control‐Faint and POTS‐Faint with rapid hypotension followed by bradycardia associated with decreased SVR and CO. This did not occur in Control‐noFaint.

**Figure 3:**
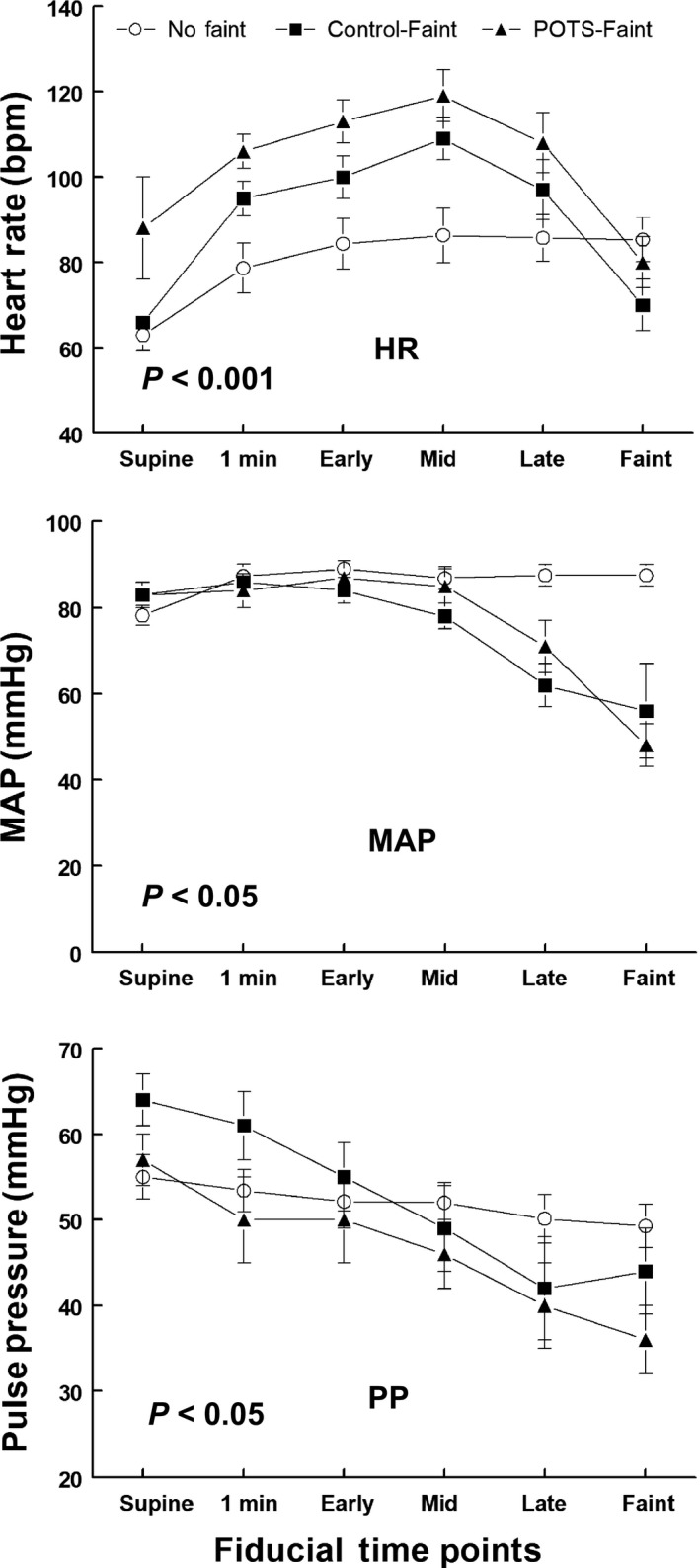
Averaged heart rate, mean arterial pressure, and pulse pressure during upright tilt. Heart rate (HR) appears in the upper panel, mean arterial pressure (MAP) in the middle panel and pulse pressure (PP) in the lower panel at designated fiducial time points. Control‐noFaint data are shown as open circles (Ο), Control‐Faint data are shown as black boxes (■), and POTS‐Faint are shown as black triangles (▲).HR was increased above control (*P* < 0.001) in all faint groups prior to faint. HR fell precipitously in all Fainters at the time of faint. MAP and PP decreased significantly throughout tilt in all VVS (*P* < 0.05) compared to Control‐noFaint subjects.

**Figure 4:**
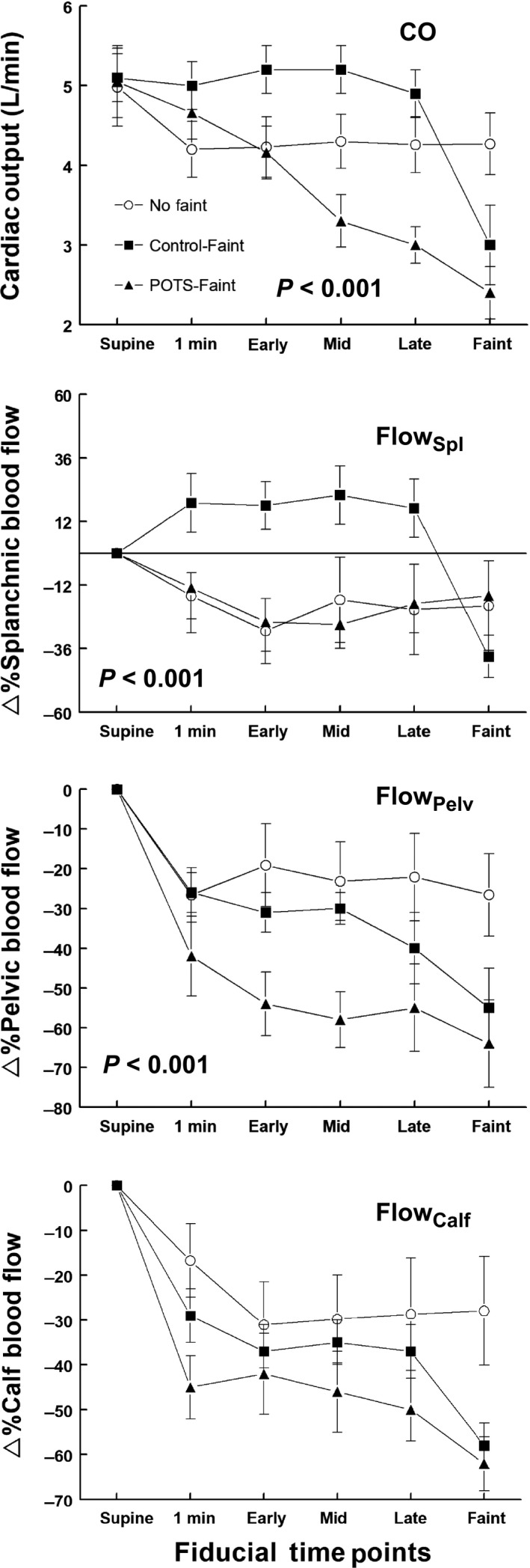
Regional blood flows during upright tilt. The top panel shows the cardiac output (CO) in L/min, the second shows percent change in splanchnic blood flow (%∆ Splanchnic Blood Flow), the third panel shows percent change in pelvic blood flow (%∆ Pelvic Blood Flow), and the bottom panel shows percent change in calf blood flow (%∆ Calf Blood Flow). Control‐noFaint data are shown as open circles (Ο), Control‐Faint data are shown as black boxes (■), POTS‐Faint are shown as black triangles (▲). CO decreased progressively in POTS‐Faint compared to Control‐noFaint (*P* < 0.001). Splanchnic blood flow was increased in Control‐Faint (*P* < 0.001).

**Figure 5:**
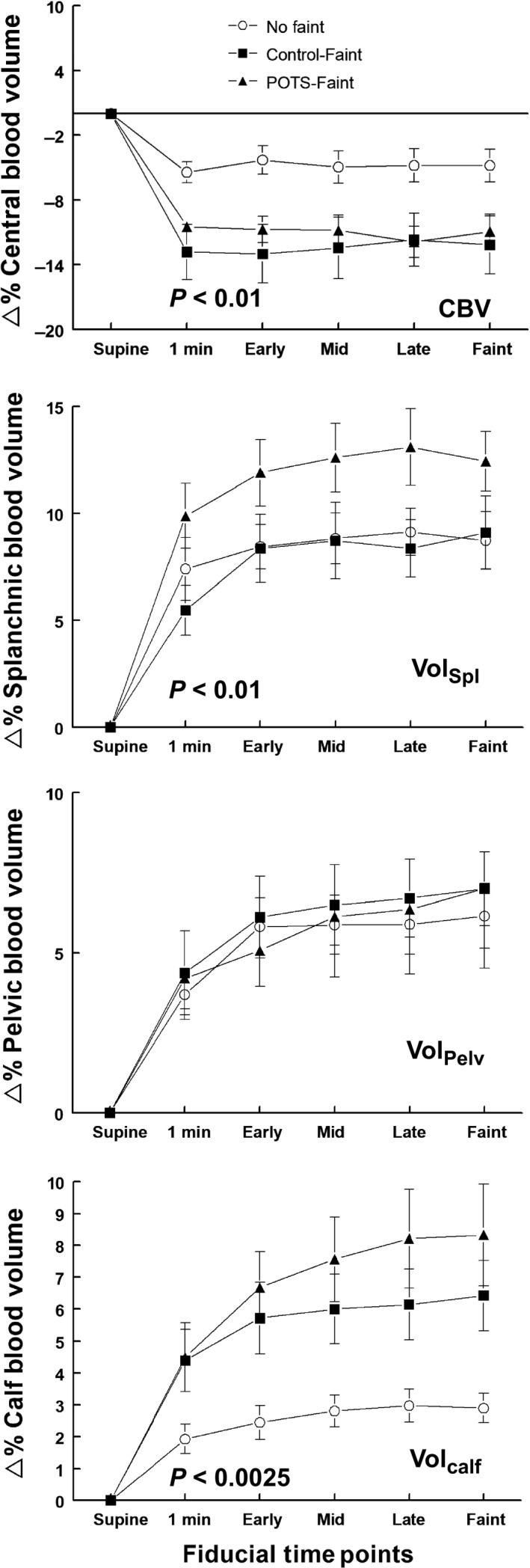
Percent change in regional blood volumes during upright tilt. The top panel shows the percent change in central blood volume (%∆ Central Blood Volume), the second shows percent change in splanchnic blood volume (%∆ Splanchnic Blood Volume), the third panel shows percent change in pelvic blood volume (%∆ Pelvic Blood Volume) and the bottom panel shows percent change in calf blood volume (%∆ Calf Blood Volume). Control‐noFaint data are shown as open circles (Ο), Control‐Faint data are shown as black boxes (■), POTS‐Faint are shown as black triangles (▲). Central*, s*planchnic, pelvic, and calf blood volumes are expressed as percent change from supine, measured before subjects were tilted upright to 70°. The Percent Central Blood Volume (%CBV) decreased in all subjects with tilt. %CBV decreased significantly more in fainting subjects than Control‐noFaint (*P* < 0.01). The Percent Splanchnic Blood Volume (Vol_Spl_) increased during tilt in all subjects, was similar to Control‐noFaint in Control‐Faint, but was significantly larger for POTS‐Faint compared to Control‐noFaint (*P* < 0.01). The Percent Pelvic Blood volume (Vol_Pelv_) increased similarly in all groups during tilt. %Calf Blood Volume increased with tilt for all subjects but was further increased in both fainting groups compared to Control‐noFaint (*P* < 0.0025).

**Figure 6:**
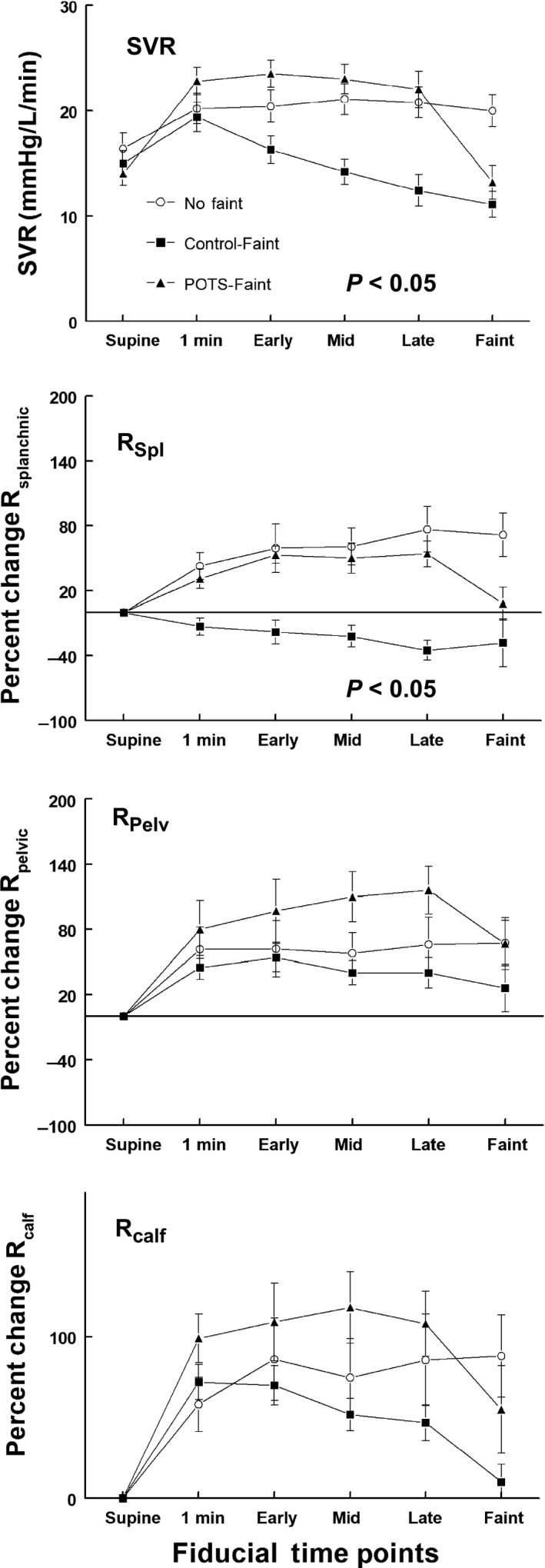
Percent change in regional arterial vascular resistance during upright tilt. The top panel shows the percent change in systemic vascular resistance (SVR), the second shows percent change in splanchnic resistance (%ΔR_Splanchnic_), the third panel shows percent change in pelvic resistance (%ΔR_Pelvic_) and the bottom panel shows percent change in calf resistance (%ΔR_calf_). Control‐noFaint data are shown as open circles (Ο), Control‐Faint data are shown as black boxes (■), POTS‐Faint are shown as black triangles (▲). SVR and %ΔR_Splanchnic_ were markedly reduced in Control‐Faint and slightly increased in POTS‐Faint. Splanchnic, pelvic, and calf blood vascular resistances are expressed as percent change from supine, measured before subjects were tilted upright to 70°. SVR increased by 1 min following tilt for all subjects. SVR then decreased progressively in Control‐Faint (*P* < 0.05). SVR remained similar to Control‐no‐Faint for POTS‐Faint SVR until syncope supervened. Splanchnic Resistance increased similarly for Control‐noFaint and POTS‐Faint until syncope occurred, while decreasing throughout tilt in Control‐Faint (*P* < 0.005).

Averaged hemodynamic data are shown in Figures 3, 4, 5, 6.

#### HR, MAP, pulse pressure (PP)

Following subjects being tilted upright to 70°, HR was increased above Control‐noFaint (*P* < 0.001) in Control‐Faint and POTS‐Faint before the “late” fiducial point when HR fell precipitously. MAP and PP were decreased significantly during tilt compared with Control‐noFaint (*P* < 0.05) (Fig. 3).

#### Regional blood flows

Splanchnic, pelvic, and calf blood flows are expressed as percent change from supine, measured before subjects were tilted upright to 70°. Control‐noFaint had an initial decrease in CO by 1 min after tilt which was sustained. With tilt CO decreased progressively in POTS‐Faint compared with Control‐noFaint (*P* < 0.001). CO did not decrease with tilt in Control‐Faint but instead was sustained at supine values Group comparison with Control‐noFaint) until syncope supervened and CO fell precipitously (Fig. 4).

Splanchnic Blood Flow decreased similarly from supine in Control‐noFaint and in POTS‐Faint. Splanchnic blood flow increased from supine in Control‐Faint (*P* < 0.001), until syncope supervened and then fell precipitously. Pelvic Blood Flow decreased with tilt in all groups and was not different in Control‐Faint compared to Control‐noFaint until syncope supervened. Pelvic blood flow was reduced during tilt in POTS‐Faint compared to Control‐noFaint (*P* < 0.001). Calf Blood Flow decreased similarly with tilt for all subjects prior to faint but decreased for both fainting groups when syncope supervened.

#### Regional blood volumes

Central*, s*planchnic, pelvic, and calf blood volumes are expressed as percent change from supine, measured before subjects were tilted upright to 70°. The Percent Central Blood Volume (%CBV) decreased in all subjects with tilt. %CBV decreased significantly more in fainting subjects than Control‐noFaint (*P* < 0.01). The Percent Splanchnic Blood Volume (Vol_Spl_) increased during tilt in all subjects, was similar to Control‐noFaint in Control‐Faint, but was significantly larger for POTS‐Faint compared to Control‐noFaint (*P* < 0.01). The percent Pelvic Blood volume (Vol_Pelv_) increased similarly in all groups during tilt. The %Calf Blood Volume increased with tilt for all subjects but was further increased in both fainting groups compared to Control‐noFaint (*P* < 0.0025) (Fig. 5).

#### Regional arterial resistances

Splanchnic, pelvic, and calf blood vascular resistances are expressed as percent change from supine, measured before subjects were tilted upright to 70°. SVR increased by 1 min following tilt for all subjects. SVR then decreased progressively in Control‐Faint (*P* < 0.05). SVR remained similar to Control‐no‐Faint for POTS‐Faint SVR until syncope supervened (Fig. 6).

Splanchnic Resistance increased similarly for Control‐noFaint and POTS‐Faint until syncope occurred, while decreasing throughout tilt in Control‐Faint (*P* < 0.005). Pelvic and calf Resistance increased in all groups and was larger than Control in POTS‐Faint until the time of syncope. Calf Resistance increased initially from supine in all subjects then decreased at the time of syncope in all fainters.

## Discussion

In this study, decreased SVR caused by impaired splanchnic arterial vasoconstriction in the absence of splanchnic pooling is the mechanism for faint in Control‐Faint. This is similar to the predominant mechanism for faint in young patients with recurrent VVS (van Steenwijk et al. 1995; Lautt 2009). Decreased CO caused by enhanced splanchnic pooling is the mechanism for faint in POTS‐Faint. This is similar to the predominant mechanism for faint in older recurrent VVS patients (Lautt 2009; Kimpinski et al. 2012).

We have previously demonstrated that decreased SVR, primarily due to splanchnic vasodilation rather than decreased cardiac output, was the main mechanism for VVS during orthostatic stress in young recurrent fainters. (Stewart et al. 2017a). We also showed that inhibiting NO synthesis increased adrenergic vasoconstriction, increased SVR and splanchnic vasodilation, and normalized orthostatic tolerance in young patients with VVS (Stewart et al. 2016, 2017b). Blood flow and vascular resistance responses to orthostatic stress in our current Control‐Faint group were similar to those found previously in recurrent VVS patients, findings caused exclusively by decreased SVR.

Our current observations contrast with those of Fu et al. (2012) who found that a minority of healthy volunteers who lacked real‐world recurrent syncope yet experienced VVS during upright tilt had decreased SVR as the mechanism for hypotension. The age range of their cohort was wider than ours, being both younger and older And studies have confirmed a progressively larger role of declining cardiac output with age in syncope in healthy volunteers (Hainsworth and Al‐Shamma 1988). Other studies of younger healthy volunteers experiencing vasovagal syncope or presyncope show maintained CO and falling SVR during orthostatic stress (Evans et al. 2001) in agreement with our present studies.

Our data also show that decreased CO, equivalent to decreased venous return, is the mechanism for fainting during upright tilt in POTS‐Faint patients, that is primarily due to venous pooling of blood within the splanchnic vasculature. Reduced venous return is consequent to excessive splanchnic blood pooling within the capacitance vessels of the liver and mesentery (Stewart et al. 2006) and to a lesser extent within the calf, but not within the lower abdominal and thigh vasculature (“pelvic segment”).

Splanchnic pooling in POTS‐Faint occurs despite sustained splanchnic vasoconstriction. One explanation for this finding would be an increase in splanchnic capacitance (decreased venoconstriction), but splanchnic venoconstriction and splanchnic arterial vasoconstriction typically occur in parallel (Pang 2001; Gelman and Mushlin 2004) and splanchnic vasoconstriction appears intact. The distributed hemodynamic properties of the splanchnic vasculature, arterial inflow resistance, venous capacitance, and outflow resistance (hepatic vascular resistance) occur at different locations. Arterial vasoconstriction occurs at the inlet of each splanchnic organ and is primarily mediated by sympathetic release of norepinephrine which causes adrenergic vasoconstriction of the vascular smooth muscle (Gelman and Mushlin 2004; Lautt 2009). In humans, splanchnic venous capacitance resides largely within the mesenteric venous system and the liver (Pang 2001; Gelman and Mushlin 2004; Lautt 2009) which are connected in series at similar pressures by the low resistance portal vein. The hepatic and mesenteric capacitance vessels have both α1‐ and α2‐adrenergic receptors contributing to active venoconstriction but lack β2 receptors (Patel et al. 1981; Rothe 1983; Gelman and Mushlin 2004). Splanchnic outflow resistance localizes mostly to the hepatic sinusoids in humans, referred to as “intrahepatic resistance” which is strongly nitric oxide (NO) dependent (Lautt 2009; Vollmar and Menger 2009). Ordinarily intrahepatic resistance is low and maintains hepatic and mesenteric capacitances at pressures only slightly higher than inferior vena cava pressure (Greenway and Lautt 1988). Small increments in intrahepatic resistance due to NO deficiency can exert large effects on hepatic and mesenteric venous pressures which may in part explain splanchnic pooling in POTS (Medow et al. 2005; Liao et al. 2010; Stewart et al. 2011).

Therefore, intrahepatic resistance is likely increased in POTS‐Faint, resulting in pooling despite sustained splanchnic arterial vasoconstriction. In addition, both intrahepatic resistance and splanchnic arterial vasoconstriction are likely reduced in Control‐Faint, resulting in increased splanchnic blood flow and reduced splanchnic resistance. Changes in NO may play a role which holds forth the potential for treating splanchnic pooling and specific disorders of orthostatic intolerance with agents that increase NO.

In summary, we found that false positive VVS in young healthy volunteers resulted from decreased systemic vascular resistance due primarily to vasoconstrictive deficits of splanchnic vasculature with sustained cardiac output. In contrast, we found that young POTS patients have increased systemic resistance and decreased venous return and cardiac output caused by excessive venous pooling in the splanchnic vasculature.

### Limitations

POTS‐Faint and Control‐Faint were identified by their fainting within 10 min of tilt. We used standard 10‐min tilt as a design feature in all our studies of POTS patients. All of our Control‐Faint patients also had 10 min studies. Increasing the time of HUT could potentially yield different information. However, the mean time to VVS in past studies of recurrent fainters was 10–11 min, and our results inform primarily on phase 2 hypotension which started before 10 min in all VVS patients.

Fiducial time markers remove the absolute time dependence of the observed phenomena while facilitating inter‐group comparison.

Impedance plethysmography of the splanchnic vasculature cannot distinguish blood pooling among the splanchnic organs (e.g., liver vs. mesenteric) and only measures fractional change in regional blood volumes and blood flows. These were validated against reference standards in prior studies (Stewart et al. 2006, 2007).

Modelflow methods yield measures of relative cardiac output. These were standardized while supine CO against inert gas rebreathing CO. Inert gas rebreathing requires deep breathing was not performed during the tilt because it would alter hemodynamics.

## Conflict of Interest

None declared.
